# Adjuvant therapy with total salvianolic acid injection enhances the safety and efficacy of recombinant prourokinase in ischemic stroke by mitigating blood–brain barrier disruption

**DOI:** 10.1186/s12967-026-08583-3

**Published:** 2026-07-14

**Authors:** Xiao-Yi Wang, Quan Li, Li Yan, Di Wang, Chun-Shui Pan, Jing-Yu Fan, Zhi-Zhong Ma, Jing-Yan Han

**Affiliations:** 1 Department of Integration of Chinese and Western Medicine, School of Basic Medical Sciences, Peking University, 38 Xueyuan Road, Beijing, 100191 People’s Republic of China; 2 Tasly Microcirculation Research Center, Peking University Health Science Center, Beijing, China; 3 Key Laboratory of Stasis and Phlegm, State Administration of Traditional Chinese Medicine of the People’s Republic of China, Beijing, China; 4 Key Laboratory of Microcirculation, State Administration of Traditional Chinese Medicine of the People’s Republic of China, Beijing, China; 5 State Key Laboratory of Core Technology in Innovative Chinese Medicine, Beijing, China; 6 China Rehabilitation Science Institute, China Rehabilitation Research Center, Beijing, China; 7 Beijing Luhe Hospital Affiliated to Capital Medical University, Beijing, China

**Keywords:** Thrombolysis, Blood-brain barrier, Ischemic stroke, Recombinant prourokinase, Salvianolic acids

## Abstract

**Background:**

Late-phase thrombolysis within the 4.5-hour window for acute ischemic stroke exacerbates blood-brain barrier (BBB) disruption, increasing the risk of hemorrhagic transformation. Recombinant human prourokinase (rhPro-UK) is an emerging thrombolytic agent, but its impact on BBB integrity compared to alteplase (recombinant tissue plasminogen activator, rt-PA) and its potential additive effect with adjuvant neuroprotectants like Total Salvianolic Acid Injection (TSI) remain unclear.

**Methods:**

A mouse thromboembolic stroke model was established, and treatments were administered intravenously 4.5 h post-occlusion. Mice were randomized into groups including sham, thrombus, rt-PA, rhPro-UK, TSI, and rhPro-UK + TSI. Cerebral blood flow, thrombus area, infarct volume, neurological scores, and BBB integrity (Evans blue leakage, cerebral edema, hemorrhage) were assessed. In vitro, human brain microvascular endothelial cells (HBMECs) underwent oxygen-glucose deprivation/reoxygenation (OGD/R) with or without rhPro-UK and TSI. Transendothelial electrical resistance, permeability, junctional proteins, matrix metalloproteinase (MMP)-2/9 activity, and oxidative stress markers were evaluated. Data were analyzed by ANOVA (Analysis of Variance) with Bonferroni post-hoc tests.

**Results:**

rhPro-UK induced less Evans blue leakage and hemorrhagic transformation than rt-PA. Combined rhPro-UK and TSI treatment, but not monotherapies, significantly reduced cerebral infarction and improved neurological function. This combination preserved BBB integrity by preventing the degradation of junctional proteins (occludin, VE-cadherin) and basement membrane components (collagen IV). In HBMECs, TSI co-treatment attenuated OGD/R+rhPro-UK-induced barrier dysfunction, suppressed reactive oxygen species (ROS) production, and reduced MMP‑2‑related gelatinolytic activity without altering MMP-2/9 expression. TSI also mitigated membrane translocation of the NADPH oxidase (NOX) subunits gp91phox and p47phox.

**Conclusions:**

rhPro-UK exhibits a superior safety profile over rt-PA regarding BBB disruption. TSI potentiates rhPro-UK-mediated protection to improve BBB integrity during late-phase thrombolysis by suppressing NOX-2-derived oxidative stress and subsequent MMP-2 activation. This combinatory strategy represents a promising therapeutic approach to enhance the safety and efficacy of delayed thrombolysis within the standard 4.5‑hour window for ischemic stroke.

**Supplementary Information:**

The online version contains supplementary material available at https://doi.org/10.1186/s12967-026-08583-3.

## Introduction

Ischemic stroke, a critical global health challenge, ranks as the second leading cause of mortality and a major contributor to long-term disability [1]. Current clinical management relies on intravenous thrombolysis, primarily using recombinant tissue plasminogen activator (rt-PA), which promotes fibrinolytic activity by converting plasminogen to plasmin. However, this therapy is constrained by a narrow 4.5-hour therapeutic window, beyond which delayed administration significantly increases risks of serious or fatal symptomatic hemorrhage [2]. The aggravated blood-brain barrier (BBB) disruption is the pathological basis of cerebral hemorrhage induced by delayed thrombolysis. The BBB functions as a specialized neurovascular system integrating neurons, astrocytes, microglia, pericytes, and endothelial cells. Its structural stability primarily arises from brain microvascular endothelial cells connected via two essential junctional assemblies: tight junctions (TJs) that restrict paracellular diffusion and adherens junctions (AJs) that ensure endothelial continuity. Disruption of junction protein complexes is a hallmark of BBB disruption in the setting of ischemic stroke [3]. Contemporary research in BBB pathophysiology has elucidated diverse molecular mechanisms capable of inducing structural and functional perturbations within cerebral microvasculature. Pharmacological interventions—including anti-inflammatory agents, matrix metalloproteinase (MMP) inhibitors, and vascular stabilizers—have demonstrated protective effects against thrombolysis-associated BBB disruption [4]. However, the inherent structural complexity of the BBB underscores the limitations of mono-therapeutic approaches in addressing multifactorial pathologies, particularly for managing hemorrhagic transformation risks exacerbated by delayed thrombolytic administration [5].

Recombinant human prourokinase (rhPro-UK), a 411-amino acid glycosylated single-chain proenzyme produced through transfected murine SP2/0 hybridoma cell technology, exhibits superior pharmacodynamic characteristics compared to alteplase in thrombolytic applications [6]. Unlike rt-PA, rhPro-UK preserves fibrinolytic specificity through a unique activation mechanism: structural reconfiguration resulting from its binding to lysine residues on fibrin Y/E fragments facilitates preferential activation of fibrin-anchored plasminogen. This process initiates a localized proteolytic cascade while maintaining hemostatic thrombus integrity, as endogenous rt-PA activity remains spatially restricted to pathological thromboembolic loci without forming covalent complexes with systemic protease inhibitors [7]. In animal stroke models, rhPro-UK enhanced recanalization rates, diminished cerebral infarct volumes, and improved neurological outcomes following intravenous administration. Notably, these therapeutic advantages coincide with significantly lower rates of systemic hemorrhagic events and reduced intracranial bleeding risk relative to traditional thrombolytic agents [8–10]. Recent multicenter clinical trials have validated these findings, establishing rhPro-UK’s non-inferiority to alteplase in early-phase acute ischemic stroke intervention while documenting substantially decreased hemorrhagic complications in moderate-to-severe stroke patients treated within the 4.5-hour therapeutic window [6, 7, 11–14]. Nevertheless, comprehensive evaluation of its impact on BBB integrity following delayed administration—particularly in direct comparison with rt-PA—remains inadequately explored.

Traditional Chinese Medicine (TCM), characterized by pleiotropic mechanisms of action involving multi-component additive effects and multi-target modulation, offers unique therapeutic advantages for stroke management. Salvianolic acids (SAs)—bioactive phenolic compounds predominantly isolated from *Salvia miltiorrhiza*—have attracted significant scientific interest due to their multimodal neurovascular protective properties, including antioxidative, anti-inflammatory, and anti-apoptotic activities [15]. Total salvianolic acid injection (TSI), a lyophilized pharmaceutical preparation designed for intravenous infusion, has received regulatory approval from the Chinese State Food and Drug Administration (Z20110011) for clinical use in post-stroke rehabilitation therapy. Our previous studies demonstrated that TSI can ameliorate ischemia-reperfusion (I/R)-induced microcirculatory dysfunction and microvascular injury in multiple organ systems, mainly by inhibiting the overproduction of reactive oxygen species (ROS) through different mechanisms [16–18]. However, TSI’s potential to attenuate thrombolysis-induced BBB damage, especially in combination with rhPro-UK during delayed treatment, has not been investigated. This study therefore addresses two critical clinical questions: first, whether rhPro-UK provides superior BBB protection compared to rt-PA when administered at the late phase of the conventional 4.5-hour therapeutic window; and second, whether adjunctive TSI therapy can further improve the safety profile of delayed rhPro-UK thrombolysis. The findings aim to provide clinically relevant evidence for optimizing thrombolytic strategies in ischemic stroke management.

## Materials and methods

### Mouse model of thromboembolic stroke

Male C57BL/6 N mice (21.0 ± 2.0 g) were obtained from the Animal Center of Peking University Health Science Center (Beijing, Certificate No. SCXK 2006-0008). All experimental protocols were approved by the Committee on the Ethics of Animal Experiments of Peking University Health Science Center (Approval No. LA2016308). Mice were housed under controlled environmental conditions (ambient temperature: 23.0 ± 2.0 °C; humidity: 55 ± 5%; 12-hour light/dark cycle) with ad libitum access to food and water.

Thromboembolic stroke induction followed established protocols [19, 20]. Briefly, mice were anesthetized via intraperitoneal injection of sodium pentobarbital (2% w/v, 45 mg/kg) and secured in a stereotaxic frame. Core body temperature was maintained at 36–37 °C using a feedback-regulated heating pad. Following a midline scalp incision, the right temporal muscle was retracted to expose the skull overlying the middle cerebral artery (MCA) bifurcation. A 1-mm diameter craniotomy was performed using a high-speed microdrill, and the dura mater was carefully excised. A microneedle, made with hematologic glass capillaries (REF 555/5; Karl Hecht KG Assistant, Germany) using a vertical puller (PC-10; Narishige, Japan), was filled with 1.5 IU/µL murine α-thrombin (Enzyme Research Laboratories, IN, USA). The thrombin solution (1.5–2.0 IU) was microinjected into the MCA over 5 min to induce localized clot formation. Successful occlusion was confirmed by sustained reduction (> 70%) in cerebral blood flow (CBF) monitored via laser speckle imaging (PeriScan PIM 3; Perimed AB, Sweden) for 60 min post-injection.

### Experimental groups and drug administration

Mice were randomized into six groups (*n* = 6/group):

Sham: Saline (i.v., left femoral vein).

Thrombus: Saline (i.v., 4.5 h post-thrombosis).

Thrombus + rt-PA: Alteplase (10 mg/kg, i.v., Boehringer Ingelheim AB, Germany).

Thrombus+rhPro-UK: Recombinant prourokinase (6 mg/kg, i.v., Tasly Pharmaceutical, Tianjin, China).

Thrombus + TSI: Total Salvianolic Acid Injection (15 mg/kg, i.v., Tasly Pharmaceutical, Tianjin, China).

Thrombus+rhPro-UK + TSI: Combined rhPro-UK (6 mg/kg, i.v.) and TSI (15 mg/kg, i.v.).

Treatments were administered via femoral vein cannulation at 4.5 h post-thrombin injection using a 10% bolus followed by 60-minute infusion. For the combination group, rhPro-UK and TSI were infused separately through left and right femoral veins. The dosage of TSI was based on our previous study showing neuroprotection at such dosage [17]. Our preliminary experiments investigating the effects of TSI on hepatic function, renal function, and coagulation function demonstrated that during this study, continuous intravenous administration of TSI via tail vein for 7 days - even at doses up to 5 times the study dose - maintained most hepatic function, renal function, and coagulation parameters within near-normal ranges. When the administered dose exceeded ten times the therapeutic dose, risks of hepatotoxicity, nephrotoxicity, and coagulation disorders were observed (Figure S3).

### Cerebral blood flow and thrombus dynamics

CBF in the MCA territory was quantified using laser speckle contrast imaging (PeriScan PIM 3; Perimed AB, Sweden) at baseline, 15 min, 4.5 h, 5.5 h, and 28.5 h post-occlusion. Thrombus area was monitored via stereomicroscopy (XTL-500, Nanjing, China).

### Assessment of neurological deficit scores, cerebral infarction, and hemorrhage

Neurological function in mice was quantitatively evaluated 24 h post-treatment using a modified Neurologic Severity Score (mNSS) according to established methodology [19]. Behavioral assessments across experimental groups were conducted by a blinded investigator following standardized operational protocols to ensure objective measurement.

Following transcardial perfusion with phosphate-buffered saline (PBS) via cardiac puncture through the left ventricle, brain tissues were immediately extracted and sectioned coronally into five 1-mm-thick slices. These sections underwent immersion in 2% 2,3,5-triphenyltetrazolium chloride (TTC) solution at 37 °C for 5 min for vital staining. Digital images were acquired using an Olympus SZ-CTV imaging system (Tokyo, Japan) and subsequently analyzed with ImageJ software (version 2.0; NIH, USA) for infarct quantification. Concurrently, hemoglobin content in affected hemispheric tissues was measured spectrophotometrically using a commercial assay kit (Cayman Chemical, Ann Arbor, MI, USA) according to manufacturer guidelines.

### Determination of cerebral microvascular injury

24 h post-intervention, mice under sodium pentobarbital anesthesia (2%, 45 mg/kg) underwent craniocerebral stabilization using a stereotaxic frame. A precision-drilled cranial window (2 × 3 mm²) exposed the pial microvasculature for observation. Following intravenous administration of FITC-conjugated albumin (50 mg/kg) via femoral catheter, real-time albumin extravasation was monitored using intravital fluorescence microscopy (BX51WT; Olympus, Japan). Extravasation indices (EI) were quantified as Ii/Iv ratios (interstitial versus venular fluorescence intensity) through digital image analysis (NIH ImageJ).

Parallel experiments employed Evans blue (2%, 4 ml/kg) administered intravenously with 3-hour circulation prior to transcardial perfusion using ice-cold saline. Rapidly harvested cerebral tissues were coronally sectioned into five 2-mm slices using a brain matrix. After macroscopic imaging, tissue homogenates in 50% trichloroacetic acid underwent overnight incubation (4 °C) followed by centrifugation (10,000×g, 30 min, 4 °C). Supernatant absorbance at 620 nm was measured using a multifunctional microplate reader (Synergy 2, Bio-Rad, Hercules, CA, USA), with Evans blue concentrations determined through standard curve interpolation.

For cerebral edema assessment, right hemispheres were weighed immediately post-extraction (wet weight) and following 72-hour desiccation at 60 °C (dry weight). Tissue water content was calculated using the formula: [(wet weight - dry weight)/wet weight] × 100%.

### Cell culture

Human brain microvascular endothelial cells (HBMECs; ScienCell, San Diego, CA, USA) were cultured in DMEM supplemented with 10% fetal bovine serum, and experiments were performed when cells reached passages 6–9. For oxygen-glucose deprivation/reoxygenation (OGD/R), cells were incubated in glucose-free medium under hypoxia (1% O₂, 5% CO₂, 94% N₂) for 4.5 h, followed by reoxygenation with or without rhPro-UK (20 µg/mL) or TSI (180 µg/mL). The optimal dosage of TSI was determined by a pre-experiment. Similar to the approach used with TSI, Vas2870 (MCE, Shanghai, China) was administered after OGD/R treatment at a concentration of 10 µM.

### Permeability assay

The in vitro endothelial permeability of HBMECs was assessed using Transwell^®^ inserts (0.4 μm pore, 12 mm diameter; Corning, MA, USA) following established protocols [21, 22]. Cells were seeded at 1 × 10⁴/insert and cultured for 72 h to achieve confluent monolayers. Post-OGD/R treatment, 200 µL of normoxic medium containing 1 mg/mL FITC-dextran was administered to the apical chamber, while 600 µL normoxic medium supplemented the basolateral compartment. After 20-minute incubation, 200 µL aliquots from basolateral chambers were collected for fluorescence quantification (excitation 490 nm/emission 520 nm) using a multifunctional microplate reader.

Prior to measurements, the apical and basolateral chamber media were replaced with fresh medium, followed by a 10-minute equilibration period at room temperature. Transendothelial electrical resistance (TEER) of HBMEC monolayers was quantified using an EVOM2 resistance meter (World Precision Instruments, Sarasota, FL, USA) equipped with sterile chopstick electrodes. Measured TEER values (Ω·cm²) were calculated by subtracting TEER values without cells.

### Immunofluorescence staining

Cerebral specimens underwent 4% paraformaldehyde fixation followed by cryoprotection through graded sucrose immersion (30% w/v). Serial 10-µm coronal sections were obtained using a cryostat (Leica CM1900, Bensheim, Germany). Primary antibodies targeting structural proteins (collagen IV, laminin) and junctional components (claudin-5, VE-cadherin, occludin; Abcam, Cambridge, MA, USA) were applied overnight at 4 °C. Following PBST washes, sections were incubated with species-matched fluorescent secondary antibodies (1:100; KPL, Gaithersburg, MD, USA) for 2 h at room temperature. Endothelial specificity was confirmed using FITC-conjugated vWF antibody (GenTex, Irvine, CA, USA), with nuclear counterstaining achieved via Hoechst 33,342 (Dojindo, Kumamoto, Japan).

For cellular analyses, HBMEC monolayers grown on chamber slides (24-well format) underwent identical fixation and immunolabeling protocols. Post-fixation PBS rinses preceded sequential antigen retrieval and blocking steps. Fluorescent signals were captured through laser scanning confocal microscopy (Leica TCS-SP5, Mannheim, Germany) under standardized acquisition parameters. Quantitative image analysis was performed using ImageJ.

### ELISA

Prior to analysis, brain tissue or cellular lysates, and conditioned media were centrifuged (12,000 × g, 10 min, 4 °C) to eliminate particulate matter. Quantification of MMP-2/9, malondialdehyde (MDA), and 8-hydroxy-2’-deoxyguanosine (8-OHdG) was performed using species-specific commercial ELISA kits (Elabscience, Wuhan, China) in strict accordance with the manufacturer’s protocols. The enzymatic activity of urokinase (uPA) under TSI treatment was evaluated using a chromogenic substrate assay kit (Abcam, Cambridge, MA, USA) as per the manufacturer’s specifications.

### Gelatin zymography

MMP-2/9 proteolytic activity was analyzed through gelatin zymography following established methodology [23]. HBMEC cultures were exposed to rhPro-UK/TSI treatments or maintained as untreated controls for 24 h. Serum-free conditioned media (0.5 mL/sample) were collected and concentrated via 10 kDa molecular weight cut-off centrifugal filters (Sartorius AG, Göttingen, Germany). Protein-normalized samples underwent electrophoresis in 8% SDS-polyacrylamide gels copolymerized with 1 mg/mL gelatin substrate. Gel development included sequential renaturation, enzymatic activation, and Coomassie staining for matrix degradation visualization.

### Measurement of reactive oxygen species

Intracellular ROS levels were assessed using 2’,7’-dichlorodihydrofluorescein diacetate (DCFH-DA; Beyotime, Shanghai, China) following the manufacturer’s protocol. Fluorescence imaging was conducted with randomly selected fields captured under a fluorescence microscope (excitation: 488 nm, emission: 525 nm). Quantitative analysis of mean fluorescence intensity per field was performed using ImageJ software.

### Western blot

Brain tissues were harvested 24 h post-treatment and homogenized in RIPA lysis buffer supplemented with 1% protease and phosphatase inhibitor cocktail. Cellular protein lysates were obtained through repeated freeze-thaw cycles, while membrane protein fractions were isolated using a specialized extraction kit (Applygen, Beijing, China). Protein quantification was performed via bicinchoninic acid (BCA) assay. Equal protein aliquots were electrophoresed on SDS-PAGE gels and subsequently transferred to polyvinylidene fluoride (PVDF) membranes. Immunoblotting was conducted using primary antibodies against β-actin, VE-cadherin, occludin, claudin-5, collagen IV, laminin, MMP-2/9, NOX-1/4 and p47phox (Abcam, Cambridge, MA, USA), followed by incubation with HRP-conjugated goat anti-rabbit secondary antibody (Cell Signaling Technology, Danvers, MA, USA). Protein signals were visualized using a ChemiDoc Touch infrared imaging system (Bio-Rad, Hercules, CA, USA) and quantified with Quantity One 4.6.2 software (Bio-Rad, Hercules, CA, USA), with normalization to β-actin expression.

### Statistical analysis

Data are expressed as mean ± SEM. Two-way ANOVA with Bonferroni post-hoc testing analyzed temporal CBF and thrombus changes. One-way ANOVA with Bonferroni correction compared intergroup differences (*P* < 0.05). All analyses were performed using GraphPad Prism 9.3.1 (GraphPad Software, San Diego, CA, USA).

## Results

### Comparative effects of delayed rhPro-UK vs. rt-PA on cerebral blood flow and thrombus Resolution

**Fig. 1:**
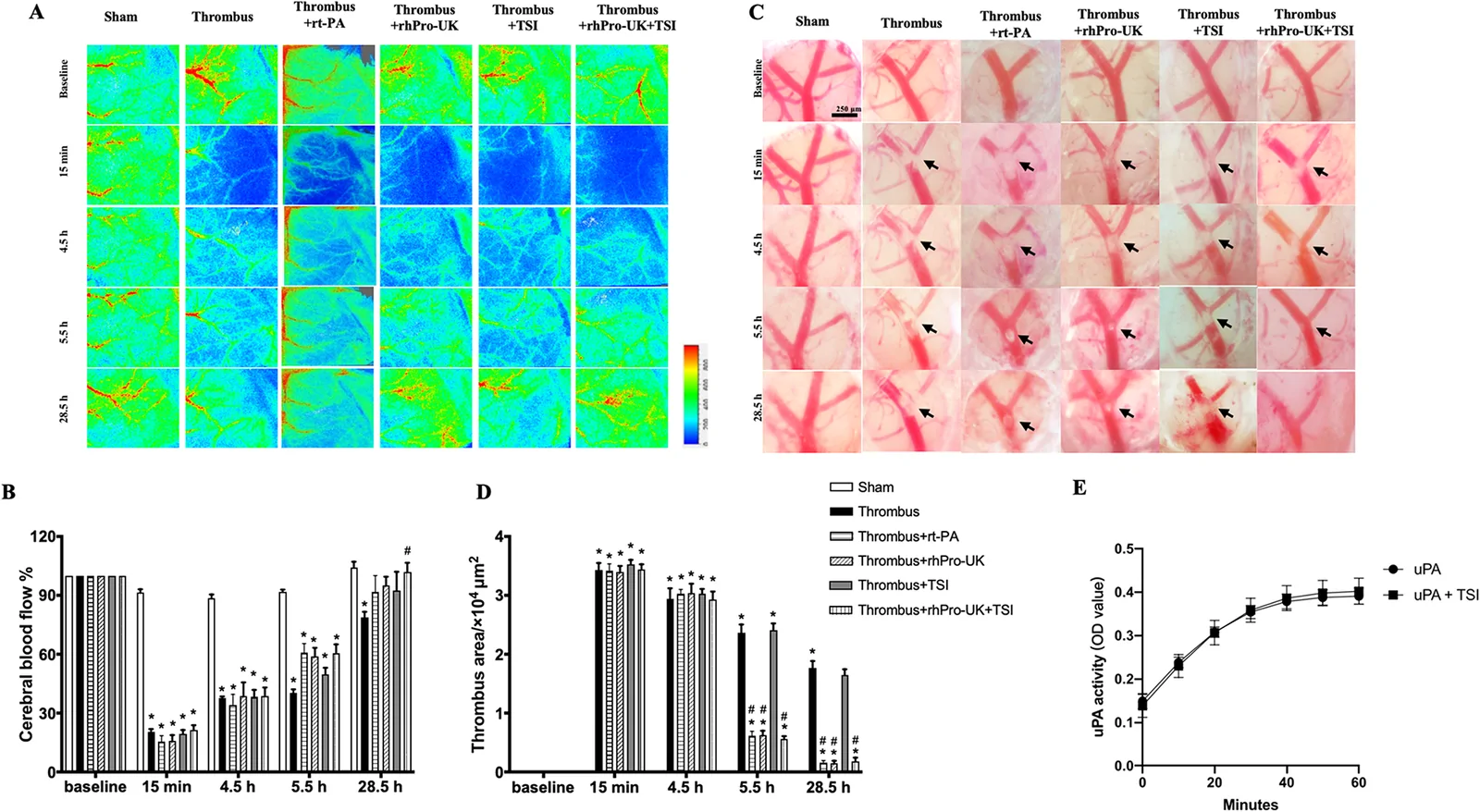
Combination of rhPro-UK and TSI promotes the recovery of cerebral blood flow**.** (**A**) Representative images of CBF at baseline, 15 min, 4.5 h, 5.5 h, and 28.5 h in every group with blue to red denoting low to high perfusion. (**B**) The statistical analysis of CBF for the above time points in mice (*N* = 6). (**C**) Representative images for dynamically observing thrombus status at baseline, 15 min, 4.5 h, 5.5 h, and 28.5 h in every group (arrows indicate thrombus. bar = 250 μm). (**D**) The statistical analysis of thrombus area for the above time points in mice (*N* = 6). (**E**) Time-dependent curve of uPA fibrinolytic activity as measured by OD value at the presence or absence of TSI. Drug administration: At 4.5 h post‑thrombosis, mice received intravenous infusions via the femoral vein as follows: rt‑PA (10 mg/kg), rhPro‑UK (6 mg/kg), TSI (15 mg/kg), or rhPro‑UK (6 mg/kg) + TSI (15 mg/kg). All treatments were given as a 10% bolus followed by a 60‑minute continuous infusion. Single agents or combinations were administered once only at the 4.5‑hour time point. ^*^P < 0.05 vs. Sham, ^#^*P* < 0.05 vs. Thrombus

CBF in the MCA territory was monitored via laser speckle contrast imaging at sequential time points (Fig. 1A). Thrombus-induced mice demonstrated significant CBF reduction post-occlusion, with residual hypoperfusion persisting until 28.5 h despite gradual temporal improvement. Delayed monotherapy with rhPro-UK or rt-PA achieved partial CBF restoration at 5.5 h post-thrombosis. Notably, combination of rhPro-UK and TSI administration significantly enhanced perfusion recovery at the 28.5-hour endpoint (Fig. 1B).

Thrombus dynamics were quantified through longitudinal MCA imaging (Fig. 1C and D). Baseline examinations confirmed patent vasculature across all cohorts. Thrombin injection induced persistent occlusive clots in the thrombus group, showing gradual dissolution over 28.5 h. Both rt-PA and rhPro-UK significantly attenuated thrombus burden compared to untreated controls, with equivalence testing confirming rhPro-UK’s non-inferiority to rt-PA. TSI exhibited no intrinsic thrombolytic activity nor modulatory effects on rhPro-UK-mediated clot resolution. Furthermore, as shown in Fig. 1E, TSI treatment did not affect uPA’s thrombolytic activity in the period of 1 h as measured periodically every 10 min. These results suggest that TSI does not affect rhPro-UK’s fibrinolytic function.

### Superior neuroprotection with combined rhPro-UK and TSI therapy

**Fig. 2:**
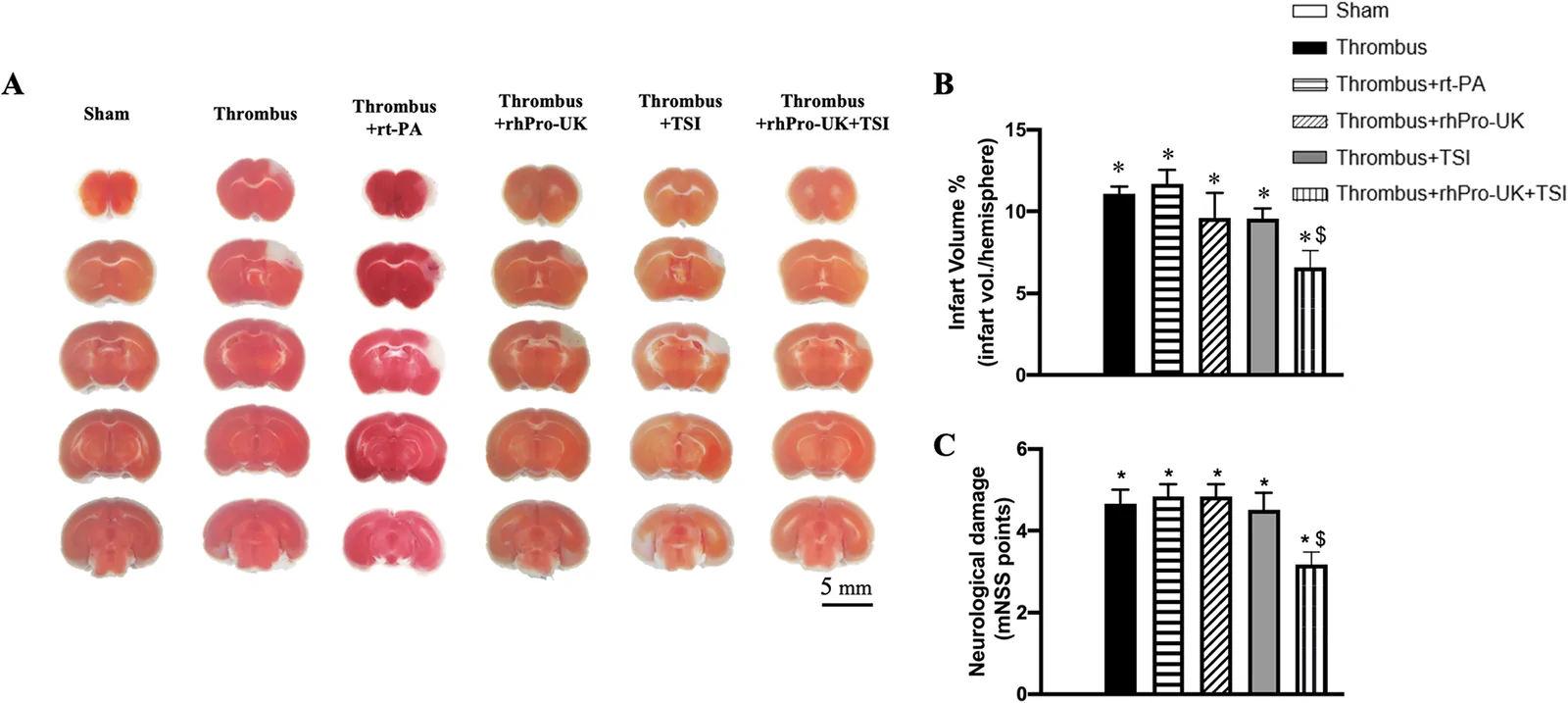
Combination of rhPro-UK and TSI attenuates infarction and improves neurological deficit. (**A**) Representative images of five coronal brain slices stained by TTC. Infarction was stained in white, while normal tissue was red. (**B**) Quantitative analysis of cerebral infarct volume (*N* = 6). (**C**) Neurological deficit scores tested with mNSS (*N* = 6). ^*^P < 0.05 vs. Sham, ^$^*P* < 0.05 vs. Thrombus+rhPro-UK

Cerebral infarction analysis revealed no pathological lesions in Sham-operated mice. Thrombus-induced mice developed extensive cortical infarcts, whereas combined rhPro-UK/TSI treatment demonstrated significant infarct attenuation. Notably, neither rt-PA nor rhPro-UK monotherapy achieved comparable neuroprotective efficacy (Fig. 2A and B). Neurological function quantified at 24 h post-treatment showed marked deficit escalation in thrombus controls versus Sham group. Monotherapy with rt-PA or rhPro-UK failed to ameliorate neurological impairments. Conversely, the rhPro-UK/TSI combinatorial regimen significantly suppressed mNSS elevation, indicating superior functional preservation compared to single-agent interventions (Fig. 2C).

### Preservation of blood-brain barrier integrity

**Fig. 3:**
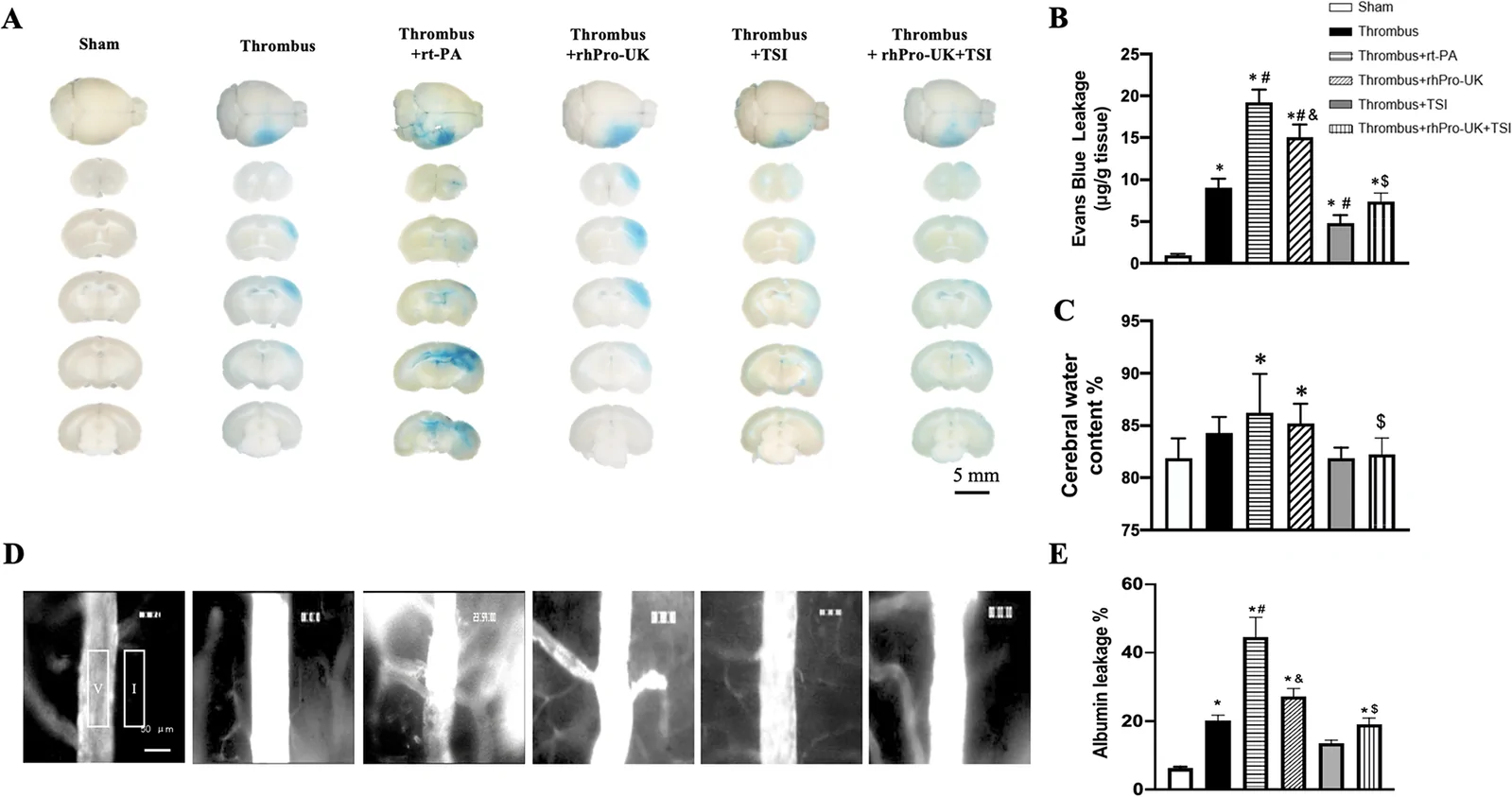
Combination of rhPro-UK and TSI reduces the BBB permeability. (**A**) Representative images of Evans blue extravasation after ischemia for 24 h in each group. (**B**) The statistical analysis of Evans blue extravasation measured by spectrophotometry (*N* = 6). (**C**) Quantitative analysis of cerebral water content in ischemic hemisphere after ischemia for 24 h in each group (*N* = 6). (**D**) Representative images of albumin leakage labeled by FITC in mouse cerebral venules. The fluorescence intensity in the venules was represented by Iv, while the interstitial area was Ii. (**E**) The statistical analysis of albumin leakage labeled by FITC (*N* = 6). ^*^P < 0.05 vs. Sham, ^#^*P* < 0.05 vs. Thrombus, ^&^*P* < 0.05 vs. Thrombus + rt-PA, ^$^*P* < 0.05 vs. Thrombus+rhPro-UK

**Fig. 4:**
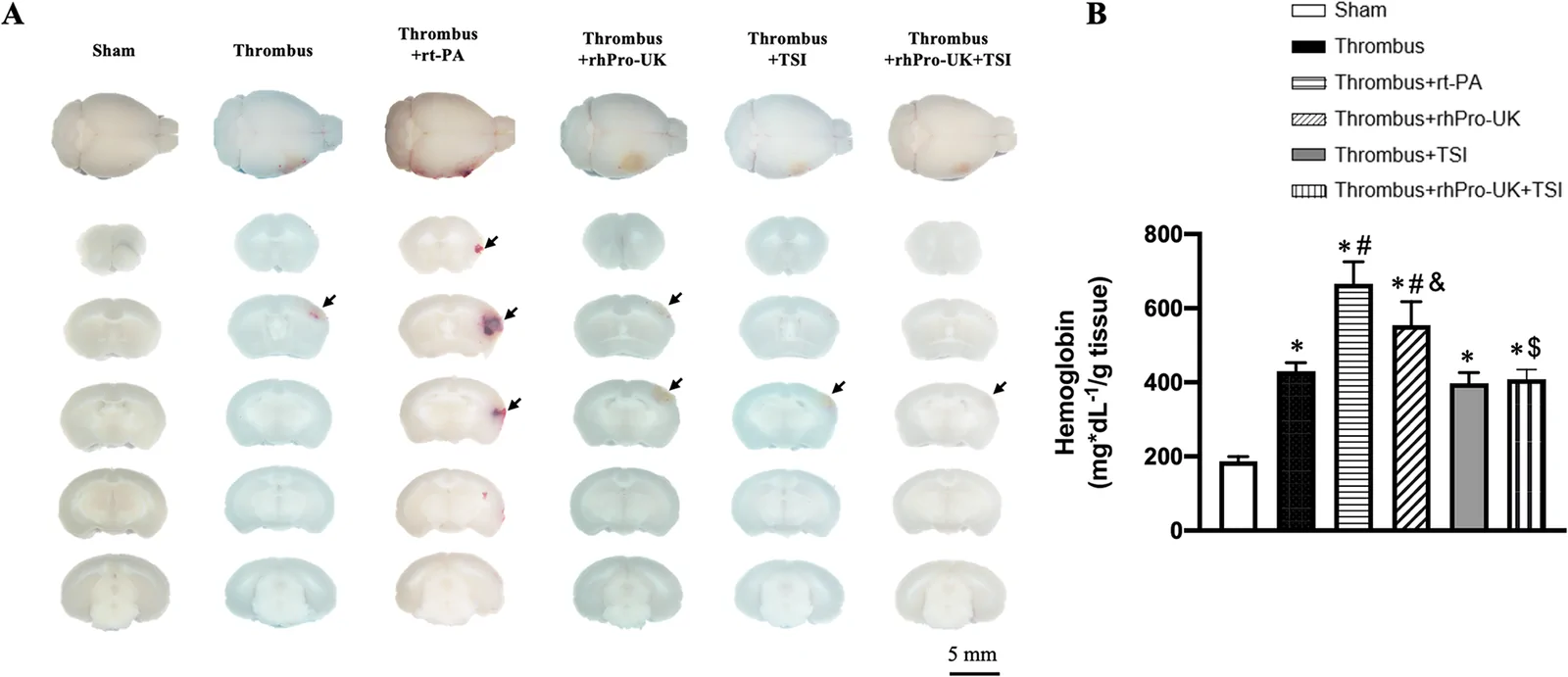
Combination of rhPro-UK and TSI decreases cerebral hemorrhage. (**A**) Representative images of whole brain and five coronal brain slices after ischemia for 24 h in each group. Arrows indicate hemorrhagic area. (**B**) Quantitative analysis of brain hemorrhage measured by colorimetric assay kit in each group. ^*^P < 0.05 vs. Sham, ^#^*P* < 0.05 vs. Thrombus, ^&^*P* < 0.05 vs. Thrombus + rt-PA, ^$^*P* < 0.05 vs. Thrombus+rhPro-UK

BBB integrity was assessed through four parameters: Evans blue extravasation, cerebral edema quantification, albumin leakage, and hemorrhagic transformation. Thrombus-induced mice exhibited significant Evans blue infiltration compared to Sham controls. Delayed rt-PA or rhPro-UK monotherapy exacerbated dye leakage in the ipsilateral hemisphere, with rhPro-UK demonstrating reduced extravasation versus rt-PA. TSI administration—both as monotherapy and combined with rhPro-UK—significantly attenuated Evans blue permeability (Fig. 3A and B). Cerebral edema analysis revealed nonsignificant water content elevation in thrombus controls relative to Sham group. Thrombolytic monotherapies markedly aggravated edema severity compared to both Sham and thrombus groups, with comparable effects between rt-PA and rhPro-UK. TSI coadministration effectively mitigated rhPro-UK-induced edema potentiation (Fig. 3C). FITC-albumin permeability assays confirmed heightened cerebrovascular leakage post-stroke. Thrombolytic interventions exacerbated albumin extravasation, though rhPro-UK showed reduced leakage versus rt-PA. TSI administration—either alone or combined with rhPro-UK—significantly suppressed albumin leakage from cerebral venule (Fig. 3D and E). Hemoglobin quantification demonstrated thrombolysis-associated hemorrhagic exacerbation. rhPro-UK demonstrated lower hemorrhage risk than rt-PA, while TSI cotreatment substantially attenuated rhPro-UK-induced cerebral hemorrhage (Fig. 4A and B). These findings demonstrate TSI’s capacity to counteract thrombolysis-related microvascular leakage and hemorrhagic complications.

### RhPro-UK and TSI additively protect endothelial junction proteins in mice

**Fig. 5:**
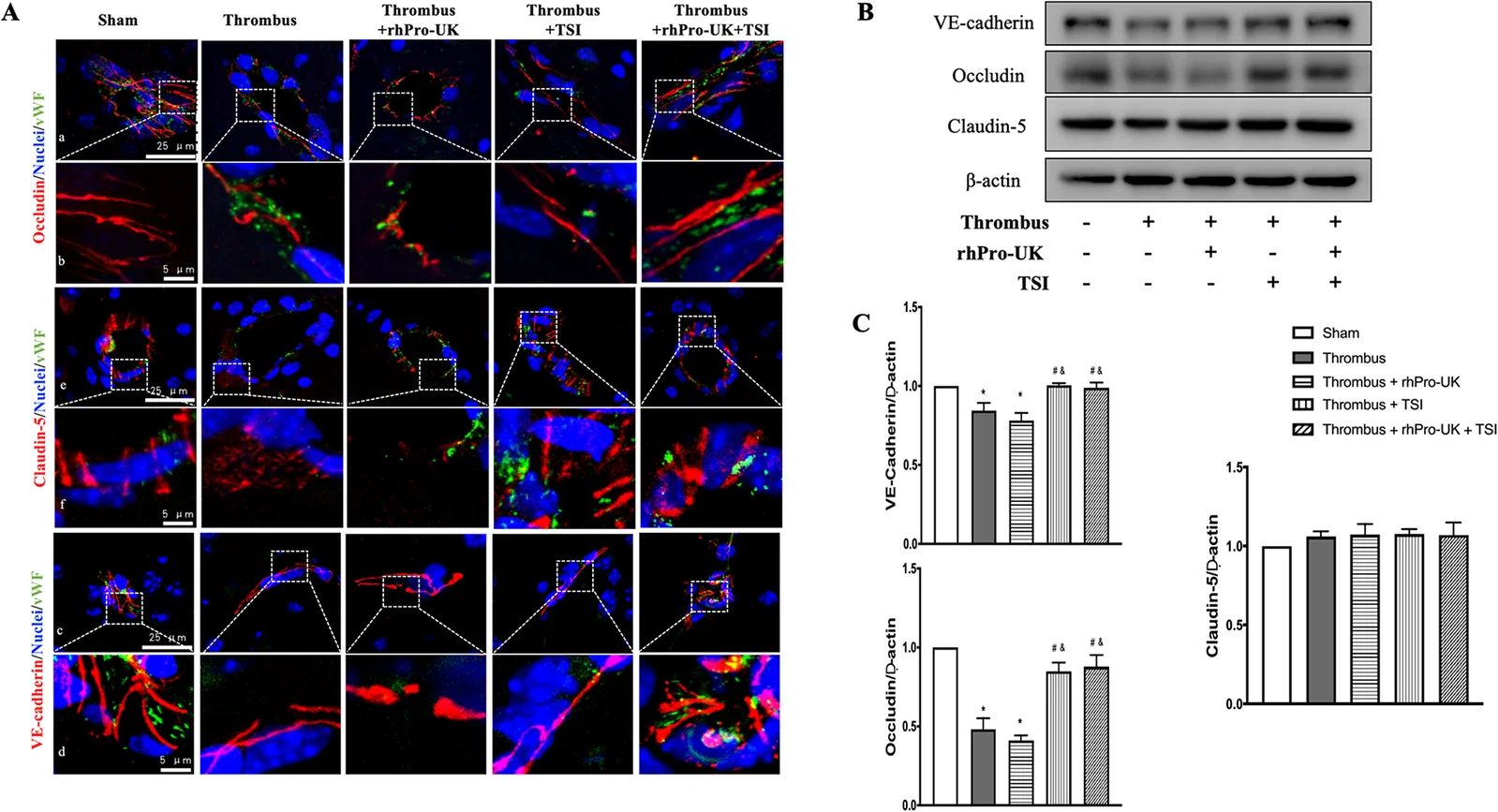
Combination of rhPro-UK and TSI maintains the integrity of endothelial cell junctions in mice. (**A**) Representative immunofluorescence images for Occludin, Claudin-5 and VE-cadherin in different group. Occludin (red), Claudin-5 (red) and VE-Cadherin (red) localized between the endothelial cell marked with vWF (green). Nuclei were stained by Hoechst (blue). (**B**) Representative images of Western blot for VE-cadherin, Occludin and Claudin-5 in ischemic hemisphere of each group. (**C**) Statistical analysis of protein levels of VE-cadherin, occludin and claudin-5 (*N* = 4). ^*^P < 0.05 vs. Sham, ^#^*P* < 0.05 vs. Thrombus, ^&^*P* < 0.05 vs. Thrombus+rhPro-UK

Immunofluorescence analysis was performed to evaluate the morphological alterations of tight junction markers (Claudin-5, Occludin) and adherens junction components (VE-Cadherin). As illustrated in Fig. 5A, sham-operated mice exhibited smooth and continuous distribution patterns of Claudin-5, Occludin, and VE-Cadherin. In contrast, the Thrombus group demonstrated reduced protein levels and discontinuous distribution of these junction proteins. Administration of rhPro-UK induced marked structural disruption, characterized by punctate fragmentation of Claudin-5, Occludin, and VE-Cadherin. Notably, co-treatment with TSI partially ameliorated the discontinuous patterns observed in the thrombus+rhPro-UK group. Western blot analysis revealed significant reductions in Occludin and VE-Cadherin protein levels in the Thrombus group compared with Sham group. The thrombus+rhPro-UK group exhibited further degradation of these proteins relative to Thrombus group. However, TSI administration effectively attenuated the rhPro-UK-induced degradation of Occludin and VE-Cadherin. No statistically significant differences in Claudin-5 protein level were detected among experimental groups (Fig. 5B and C**)**.

### RhPro-UK and TSI exert additive effects in mitigating basement membrane perturbations in mice

**Fig. 6:**
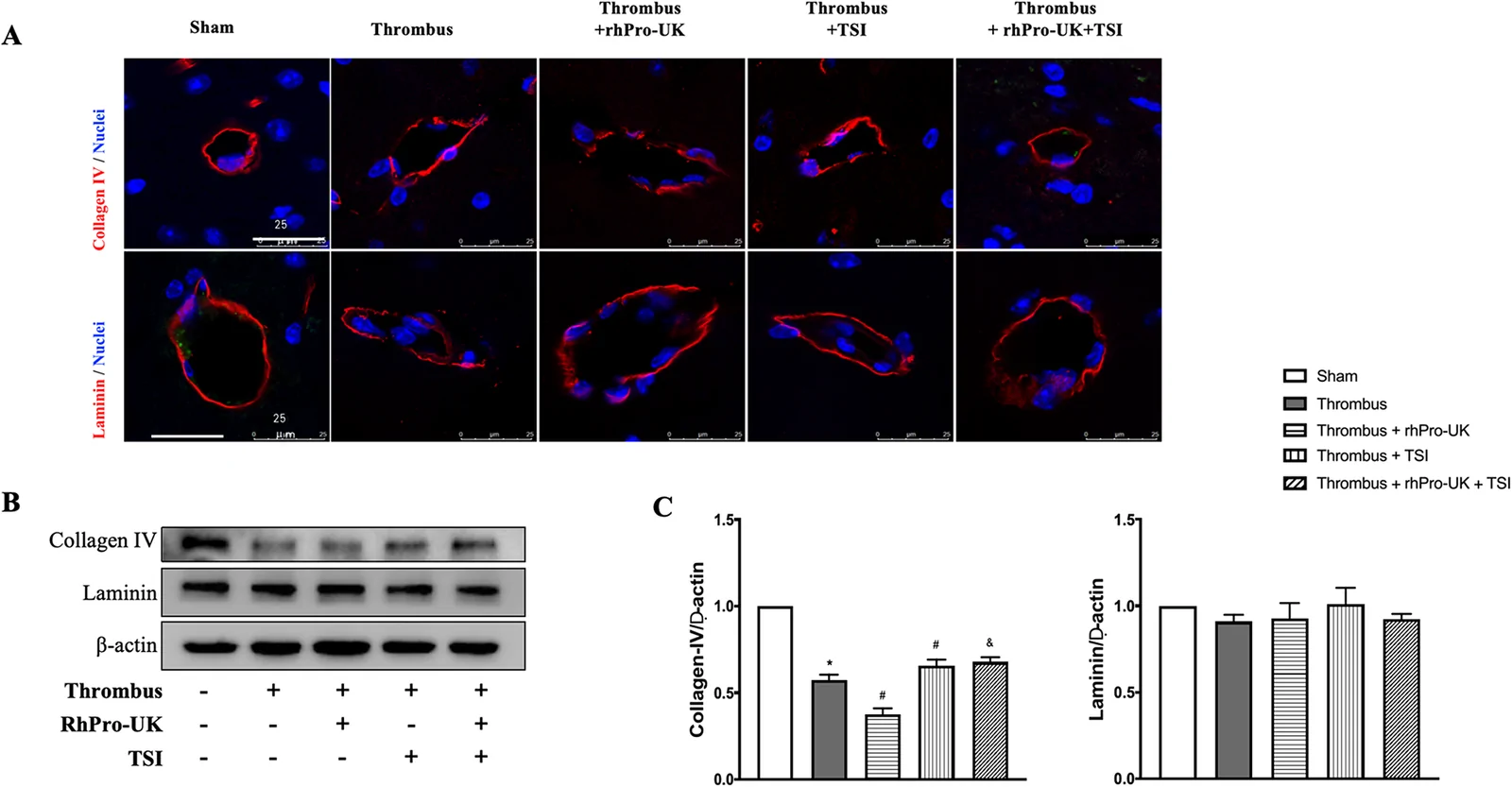
Combination of rhPro-UK and TSI mitigates basement membrane damage. (**A**) Representative immunofluorescence images for Collagen IV and Laminin in different group. Collagen IV and Laminin were stained red and nuclei was blue. (**B**) Representative images of Western blot for Collagen IV and Laminin in ischemic hemisphere of each group. (**C**) Statistical analysis of protein levels of Collagen IV and Laminin (*N* = 4). *P < 0.05 vs. Sham, #*P* < 0.05 vs. Thrombus, &*P* < 0.05 vs. Thrombus+rhPro-UK

To assess vascular wall integrity, the spatial organization of basement membrane proteins was analyzed through immunofluorescence microscopy, with particular focus on Collagen IV and Laminin distributions. As shown in Fig. 6A, sham-operated animals displayed continuous linear patterns of Collagen IV, whereas the Thrombus group exhibited fragmented Collagen IV distribution with reduced fluorescence intensity. rhPro-UK administration markedly exacerbated these structural perturbations, converting Collagen IV arrangements into discontinuous punctate formations. Notably, TSI co-treatment partially restored morphological continuity compared to the Thrombus+rhPro-UK group. Laminin distribution patterns remained comparable across all experimental conditions. Western blot quantification corroborated these morphological findings, revealing significantly decreased Collagen IV protein levels in the Thrombus group compared to Sham group. The thrombus+rhPro-UK group showed further degradation of Collagen IV relative to the Thrombus group. Importantly, TSI intervention effectively mitigated rhPro-UK-induced Collagen IV degradation. No statistically significant differences in Laminin protein level were detected across experimental groups (Fig. 6B and C).

### TSI ameliorates endothelial barrier function in vitro

**Fig. 7:**
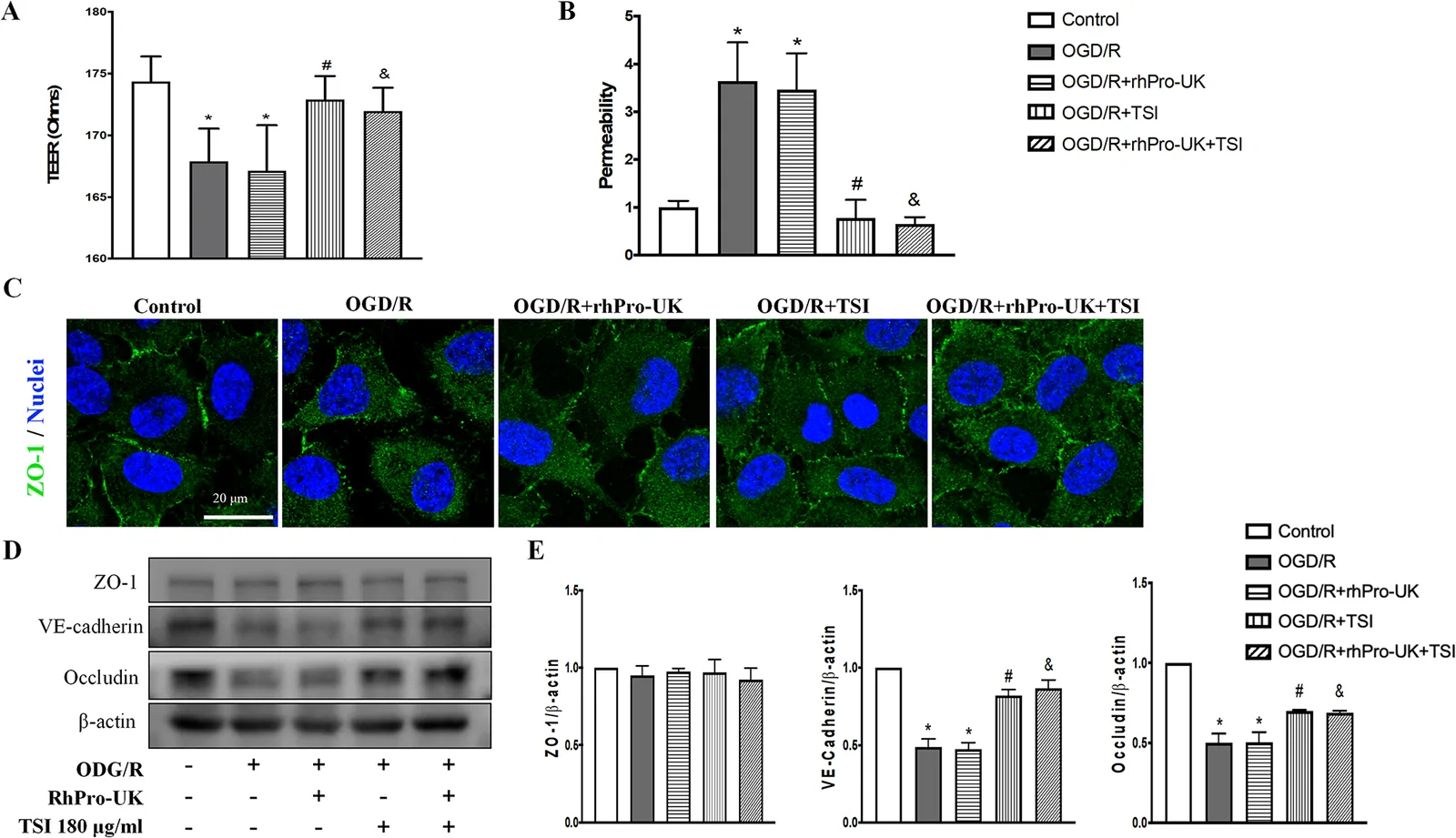
TSI ameliorates endothelial barrier function in vitro**.** The quantitative analysis of TEER (**A**) and permeability (**B**) of HBMECs following OGD/R treatment for 24 h (*N* = 3–5). (**C**) Representative immunofluorescence images for ZO-1 in different group. ZO-1 was stained red and nuclei was blue. (**D**) Representative images of Western blot for ZO-1, VE-cadherin and Occludin. (**E**) Statistical analysis of protein levels of ZO-1, VE-cadherin and Occludin (*N* = 4). ^*^P < 0.05 vs. Control, ^#^*P* < 0.05 vs. OGD/R, ^&^*P* < 0.05 vs. OGD/R+rhPro-UK

We have first measured the TEER of endothelial cell monolayers by positioning electrodes in the apical chamber (containing endothelial cells) and basolateral chamber using a resistance meter. As shown in Fig. 7A, OGD/R markedly decreased TEER in HBMEC monolayers, while rhPro-UK administration exacerbated this reduction. Conversely, TSI co-treatment significantly attenuated the OGD/R-induced TEER decline. Employing an identical experimental paradigm, FITC-dextran permeability across HBMEC monolayers in Transwell^®^ inserts was assessed. OGD/R substantially enhanced FITC-dextran permeability, an effect further amplified by rhPro-UK treatment. Notably, 24-hour TSI co-administration demonstrated a potent inhibitory effect on OGD/R-induced permeability elevation (Fig. 7B). We have then evaluated the effect of TSI on the presence of ZO-1 between endothelial cells by immunofluorescence after 24 h of treatment. As illustrated in Fig. 7C, OGD/R induced discontinuous ZO-1 localization, while rhPro-UK treatment exacerbated structural disorganization. TSI co-administration partially restored ZO-1 continuity. Further, we have also evaluated the protein levels of ZO-1, occludin and VE-cadherin by Western-blot. Our results demonstrated marked reductions in Occludin and VE-Cadherin protein levels following OGD/R compared to Control, with further protein degradation observed in OGD/R+rhPro-UK group. TSI intervention counteracted rhPro-UK-induced proteolytic effects on both adhesion molecules. ZO-1 protein level remained statistically comparable across all experimental conditions (Fig. 7D and E).

### TSI modulates MMP-2 secretion and extracellular activityTSI modulates MM

**Fig. 8:**
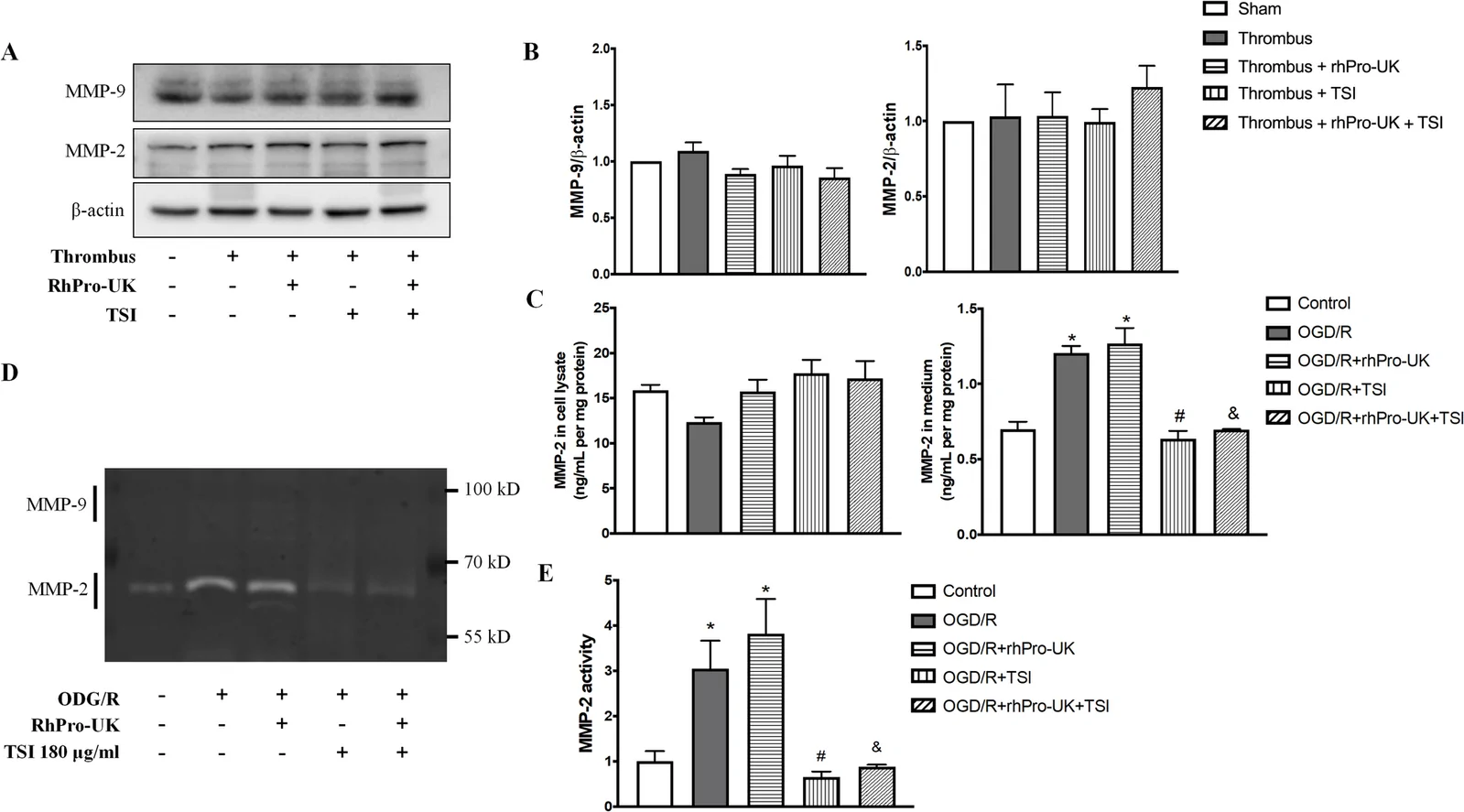
TSI modulates MMP‑2‑related gelatinolytic activity and extracellular protein levels**.** (**A**) Representative images of Western blot for MMP-9 and MMP-2 in ischemic hemisphere of each group. (**B**) Statistical analysis of protein levels of MMP-9 and MMP-2 (*N* = 6). (**C**) Quantitative analysis of MMP-2 content in HBMECs lysates and conditioned media collected from HBMECs in different group (*N* = 4). (**D**) Representative gelatin zymography gels of conditioned media collected from HBMECs in different group. (**E**) Quantitative results of MMP‑2‑related gelatinolytic activity in different group by inverse densiometric analysis (*N* = 4). ^*^P < 0.05 vs. Control, ^#^*P* < 0.05 vs. OGD/R, ^&^*P* < 0.05 vs. OGD/R+rhPro-UK

Matrix metalloproteinases mediate proteolytic degradation of junctional and basement membrane components. Western blot analysis showed comparable MMP-2 and MMP-9 protein levels across experimental groups in mice model (Fig. 8A and B). Consistent with in vivo findings, no MMP-9 differences were observed following in vitro OGD/R injury (data not shown). ELISA analysis of HBMEC lysates revealed no significant intergroup difference in MMP-2 levels (Fig. 8C). However, quantification of conditioned media from HBMECs demonstrated OGD/R-induced MMP-2 elevation that remained unaltered by rhPro-UK monotherapy. Both TSI monotherapy and its combination with rhPro-UK significantly reduced extracellular MMP-2 accumulation compared to OGD/R and rhPro-UK groups (Fig. 8C). Zymography of conditioned media detected two distinct bands at ≈ 93 kDa (MMP-9) and 65 kDa (MMP-2) (Fig. 8D). Following in vitro OGD/R injury, extracellular HBMEC-derived MMP-9 activity was barely detectable across all groups. Notably, OGD/R significantly enhanced MMP‑2‑related gelatinolytic activity compared to controls, with rhPro-UK treatment further augmenting this activity. This thrombolysis-associated increase in MMP‑2 gelatinolytic activity was effectively mitigated by TSI coadministration (Fig. 8E).

### TSI suppresses oxidative stress

**Fig. 9:**
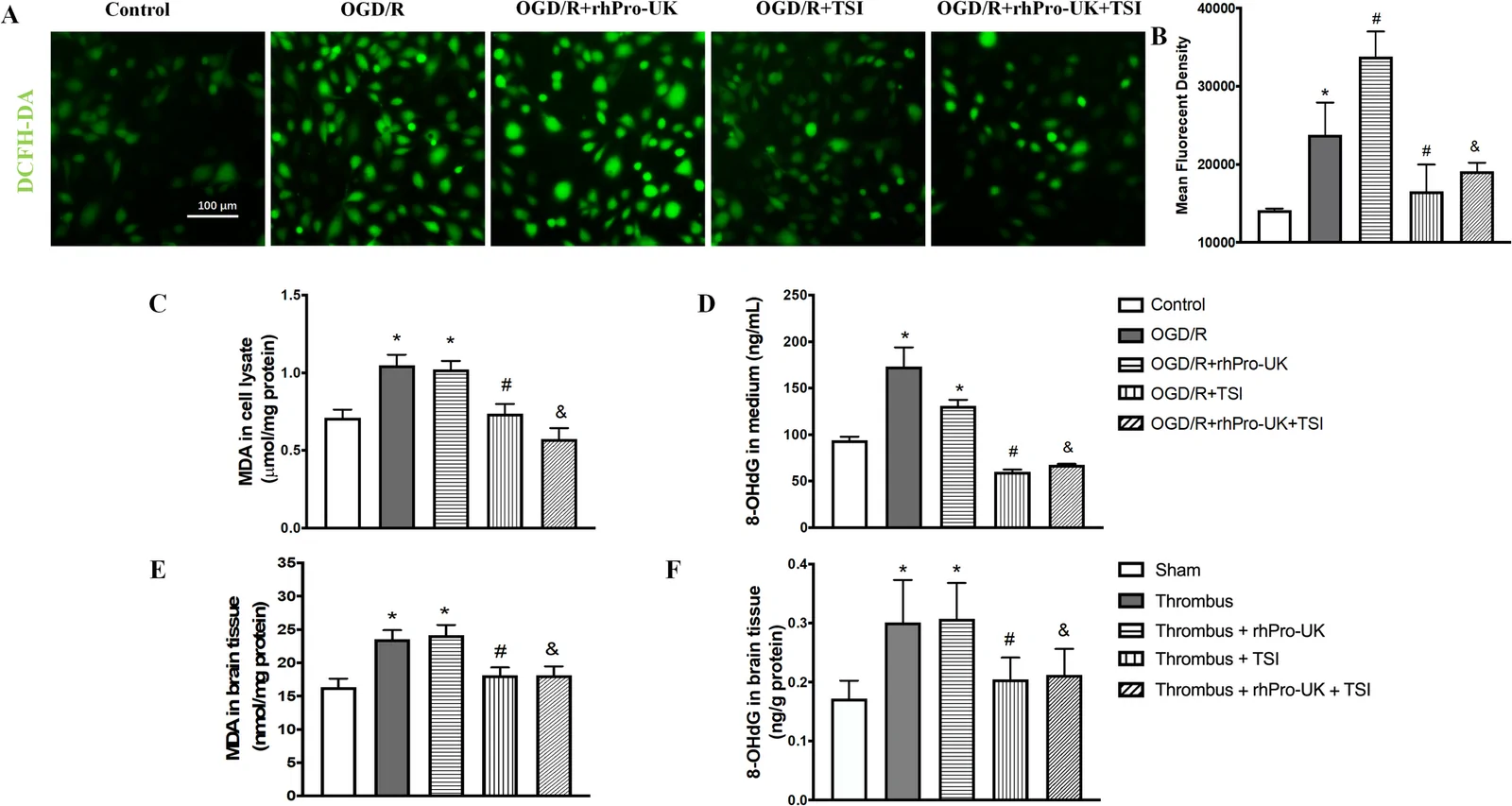
TSI suppresses oxidative stress. (**A**) Representative DCFH-DA fluorescent images in HBMECs from different group. (**B**) Quantitative results of mean fluorescence intensity of DCFH-DA in HBMECs from different group (*N* = 3). (**C**, **D**) Quantitative analysis of MDA content in HBMECs lysates (**C**) and 8-OHdG content in medium (**D**) from different group (*N* = 4). (**E**, **F**) Quantitative results of MDA (**E**) and 8-OHdG (**F**) content in ischemic hemisphere of each group (*N* = 6). ^*^P < 0.05 vs. Control, ^#^*P* < 0.05 vs. OGD/R or Thrombus, ^&^*P* < 0.05 vs. OGD/R+rhPro-UK or Thrombus+ rhPro-UK

Regarding oxidative stress evaluation, DCFH-DA staining showed that OGD/R significantly increased ROS fluorescence in HBMECs compared to controls, with rhPro-UK treatment further elevating ROS levels. TSI administration markedly attenuated this ROS fluorescence enhancement (Fig. 9A and B). ELISA analysis paralleled these findings: OGD/R and rhPro-UK significantly increased MDA content in HBMEC lysates (Fig. 9C) and 8-OHdG levels in conditioned media collected from HBMECs (Fig. 9D), both of which were substantially reduced by TSI intervention. In vivo ELISA results exhibited consistent trends with the in vitro observations (Fig. 9E and F).

### TSI modulates NADPH oxidase subunit trafficking

**Fig. 10:**
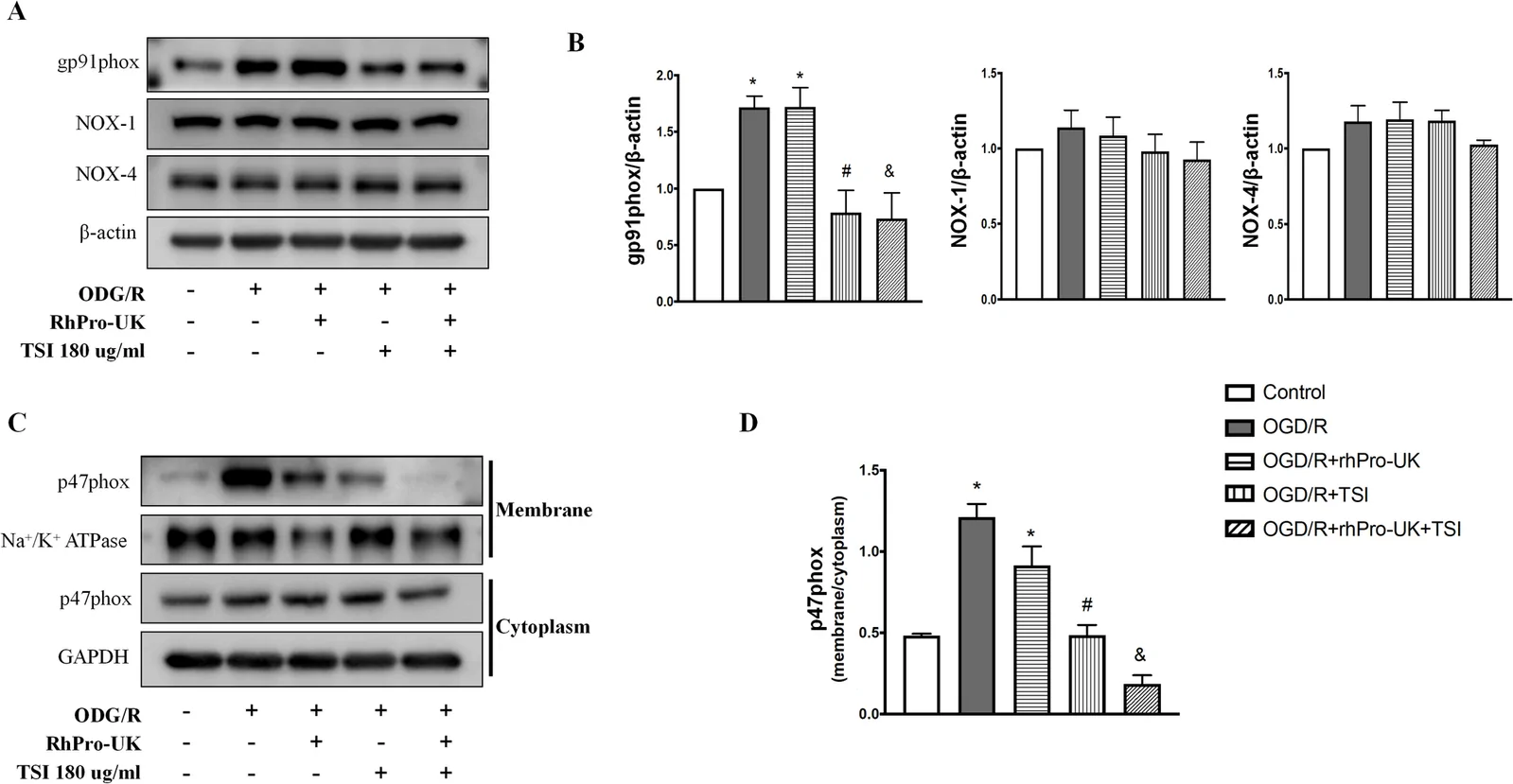
TSI treatment reduced the expression of gp91phox and attenuated membrane translocation of p47phox following OGD/R treatment for 24 h.(**A**) Representative images of Western blot for gp91phox, NOX-1 and NOX-4 in HBMECs lysates from different group. (**B**) Statistical analysis of protein levels of gp91phox, NOX-1 and NOX-4 (*N* = 4). (**C**) Representative images of Western blot for p47phox in membrane and cytoplasm of HBMECs from different group. (**D**) Statistical analysis of the ratio of p47phox protein levels in membrane and cytoplasm of HBMECs from different group (*N* = 4). ^*^P < 0.05 vs. Control, ^#^*P* < 0.05 vs. OGD/R, ^&^*P* < 0.05 vs. OGD/R+rhPro-UK

Western blot analysis in Fig. 10A and D showed the expression profiles of NOX-1, NOX-4, and NOX-2 subunits (gp91phox and p47phox) in cytosolic and membrane fractions, with corresponding quantitative analysis presented below. NOX-1 and NOX-4 expression remained consistent across treatment groups. Compared to controls, OGD/R significantly elevated the NOX-2. In contrast, cytosolic p47phox levels showed no intergroup variation, while membrane-associated p47phox increased markedly in the OGD/R group (Fig. 10C). TSI treatment effectively mitigated the OGD/R-induced membrane accumulation of both gp91phox and p47phox.

### The NOX-2 specific inhibitor Vas2870 mimics effects of TSI

**Fig. 11:**
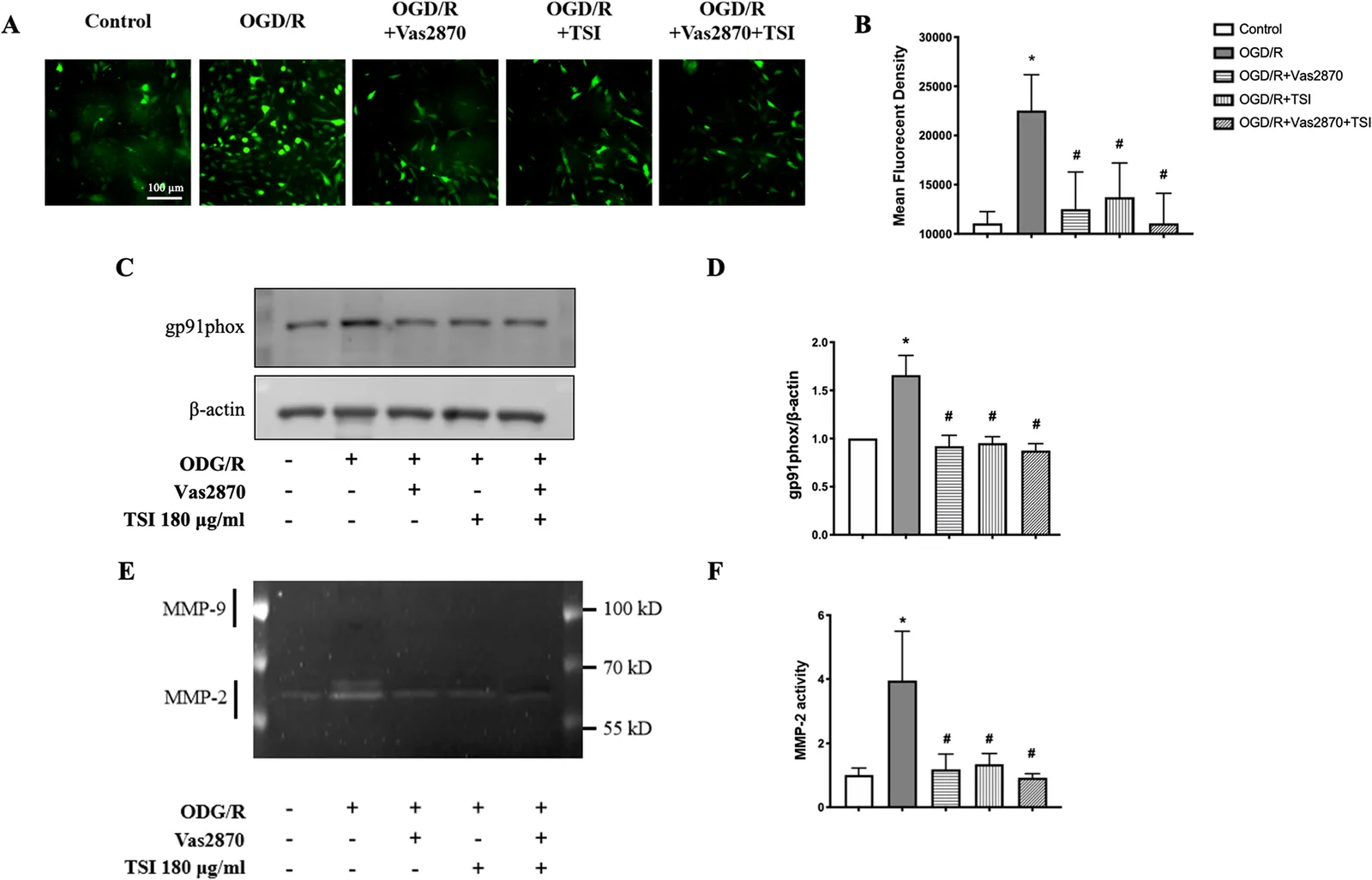
The NOX-2 specific inhibitor Vas2870 mimics effects of TSI.(**A**) Representative DCFH-DA fluorescent images in HBMECs from different group. (**B**) Quantitative results of mean fluorescence intensity of DCFH-DA in HBMECs from different group (*N* = 3). (**C**) Representative images of Western blot for gp91phox in HBMECs lysates from different group. (*N* = 4). (**D**) Statistical analysis of protein levels of gp91phox (*N* = 4). (**E**) Representative gelatin zymography gels of conditioned media collected from HBMECs in different group. (**F**) Quantitative results of MMP‑2‑related gelatinolytic activity in different group by inverse densiometric analysis (*N* = 4). ^*^P < 0.05 vs. Control, ^#^*P* < 0.05 vs. OGD/R

The NOX-2 specific inhibitor Vas2870 significantly reduced OGD/R-induced ROS generation (Fig. 11A and B), gp91phox overexpression (Fig. 11C and D), and elevated MMP-2 activity (Fig. 11E and F), with effects comparable to those of TSI. However, when TSI was combined with Vas2870, no further significant reduction in gp91phox expression or ROS generation was observed, suggesting that the mechanism of action of TSI may be closely related to NOX-2 inhibition.

## Discussion

This study provides compelling evidence that delayed thrombolysis with rhPro-UK, while exhibiting a comparatively safer profile than rt-PA, still induces significant secondary injury in a murine model of ischemic stroke, including cerebral microvascular albumin leakage, edema formation, and hemorrhagic transformation. Critically, co-administration of TSI substantially attenuated these pathological changes, concurrently reducing infarct volume and improving neurological outcomes. Mechanistically, we demonstrated that TSI confers protection to BBB integrity primarily through inhibiting p47phox membrane translocation and subsequent NOX-2 complex assembly, thereby suppressing ROS overgeneration. This reduction in oxidative stress subsequently leads to decreased MMP-2 activation, which in turn helps preserve the structural and functional integrity of endothelial junctional complexes and the basement membrane.

Recombinant tissue plasminogen activator (rt‑PA) remains the gold-standard thrombolytic agent for acute ischemic stroke, endorsed by international treatment guidelines. However, its clinical utility is constrained by three principal limitations: a narrow therapeutic window (≤ 4.5 h post-stroke onset), a significant risk of hemorrhagic transformation—particularly symptomatic intracranial hemorrhage—and considerable cost, especially in resource-limited healthcare settings [24]. These limitations have motivated the search for alternative thrombolytics with improved safety and cost-effectiveness profiles. rhPro-UK, a single-chain zymogen plasminogen activator, demonstrates a fibrin-specific activation mechanism whereby it is converted to its active two-chain form by plasmin preferentially localized on the thrombus surface [25]. This theoretically restricts fibrinolytic activity to the pathological clot, minimizing systemic plasminogen activation and thereby reducing hemorrhagic complications. Consistent with previous reports [8–10] and our preliminary experiments, we conducted a comparative thrombolytic study of rt‑PA and rhPro‑UK administered 1.5 h after thrombus formation. The results demonstrated that both agents exhibited favorable thrombolytic efficacy at this time point, with no significant differences in cerebral infarction volume ratio or hemorrhagic status (Figure S2). These findings are consistent with the conclusions reported in a previous study [9]. Therefore, we did not conduct a more in‑depth comparison of the therapeutic efficacy of these two drugs at this specific time point. In turn, our data confirmed that when thrombolytic therapy with rt‑PA and rhPro‑UK was administered 4.5 h after thrombus formation, rhPro‑UK was non‑inferior to rt‑PA in achieving vascular recanalization and reducing thrombotic burden. Crucially, administration at 4.5 h post‑thrombus formation associated rhPro‑UK with a lower risk of hemorrhagic transformation.

Nevertheless, our results unequivocally demonstrate that even this more selective thrombolytic agent, when delivered in a delayed fashion, significantly aggravates BBB permeability, cerebral edema, and albumin extravasation compared to non-thrombolytic controls. This finding underscores a critical, often underappreciated, reality: the pathophysiological cascade initiated by prolonged ischemia—including endothelial cell activation, inflammatory signaling, and oxidative stress—primes the neurovasculature for injury upon reperfusion, irrespective of the thrombolytic agent’s specificity. The process of reperfusion injury can itself unleash a torrent of damaging mediators that compromise vascular integrity.

The principal finding of this study is that TSI, a standardized preparation of salvianolic acids from *Salvia miltiorrhiza*, effectively counteracts these deleterious effects of delayed rhPro-UK thrombolysis. The combinatorial therapy not only enhanced the recovery of cerebral blood flow at later time points but also significantly reduced infarct volume and improved neurological outcomes—benefits not attained by either rhPro-UK or rt-PA monotherapy. This suggests that TSI provides a distinct neuroprotective effect that is additive to the revascularization achieved by thrombolysis. In our experimental protocol, rhPro-UK and TSI were administered concurrently as a 10% bolus followed by a 60‑minute continuous infusion via separate femoral veins starting at 4.5 h post‑thrombosis. This simultaneous delivery mirrors a realistic clinical scenario where both agents would be initiated together upon the decision to perform delayed thrombolysis. Importantly, as shown in Fig. 1E, TSI does not affect the fibrinolytic activity of uPA, the active form of prourokinase, indicating that TSI does not interfere with rhPro‑UK‑mediated clot dissolution. Conversely, TSI exerts its protective effects on the BBB by suppressing NOX-2‑derived oxidative stress and subsequent MMP‑2‑related gelatinolytic activity, pathways that are not directly targeted by rhPro‑UK. Therefore, the two agents function concurrently through parallel, non‑antagonistic mechanisms: rhPro‑UK restores perfusion by lysing the thrombus, while TSI independently protects the neurovascular unit from reperfusion‑induced injury. This concurrent action explains the additive benefits observed in the combination group without compromising thrombolytic efficacy or increasing hemorrhagic risk.

Delving into the mechanisms, we focused on the dynamic interplay between oxidative stress and MMP activation, a well-established axis in BBB breakdown. Matrix metalloproteinases (MMPs), especially MMP-2 and MMP-9, serve as pivotal mediators of endothelial dysfunction and vascular instability in the context of delayed thrombolysis for ischemic stroke [26]. During the acute phase of cerebral ischemia, upregulation of MMP-2/9 facilitates proteolytic degradation of tight junction proteins (e.g., occludin and claudin-5) and basal lamina constituents (such as collagen IV and laminin), leading to BBB breakdown, progression of cerebral edema, hemorrhagic transformation, and neuronal apoptosis [27]. The vascular basement membrane, enriched in collagen IV and laminin, constitutes a critical structural element for microvascular integrity and represents a key substrate for MMP-mediated degradation, underlying the pathogenesis of secondary hemorrhage [28]. Of clinical relevance, delayed thrombolytic treatment has been shown to elevate both the expression and proteolytic activity of MMPs in cerebral tissue through multiple pathophysiological pathways, thereby aggravating neurovascular injury during the acute stroke phase [26]. Consistent with this concept, our data indicate that rhPro-UK administration enhances OGD/R-induced MMP-2 secretion and enzymatic activity in conditioned media from HBMECs. Importantly, TSI co-treatment selectively suppressed MMP‑2‑related gelatinolytic activity without altering MMP-2/9 expression levels, while concurrently preserving key neurovascular components including endothelial junctional proteins (VE-cadherin and occludin) and basement membrane collagen IV.

Under pathological conditions such as I/R or inflammation, excessive ROS production potentiates MMP-2 activity through multi-tiered regulatory mechanisms. Oxidative stress can induce transcriptional upregulation of MMP-2 via several signaling pathways. At the post-translational level, ROS oxidize cysteine residues within the pro-domain of pro-MMP-2, facilitating its proteolytic activation. Simultaneously, ROS-induced lipid peroxidation damages cellular membranes, promoting the release of MMP-2 from intracellular stores [29]. This redox-mediated amplification of MMP-2 activity aggravates degradation of the extracellular matrix, particularly affecting collagen IV-rich basement membranes, thereby contributing to cerebral hemorrhage and edema after delayed thrombolysis in stroke [30]. Our experimental models demonstrated that rhPro-UK further exacerbated I/R-induced oxidative stress, as indicated by elevated levels of ROS, MDA, and 8-OHdG. TSI treatment effectively suppressed ROS accumulation, suggesting its capacity to reduce MMP‑2‑related gelatinolytic activity through redox modulation—evidenced by reduced gelatinolytic activity despite unaltered MMP-2 expression.

The NOX family represents a major enzymatic source of ROS and plays a central role in redox signaling under pathological conditions. In endothelial cells, NOX-2—a multi-subunit complex requiring membrane-bound p22-phox and cytosolic components (p47phox, p67phox, Rac GTPase)—is a key driver of oxidative stress upon activation. Pathological stimuli trigger translocation of cytosolic subunits to the membrane, assembling the functional holoenzyme that generates superoxide (O_2_⁻) via NADPH oxidation. Enhanced NOX-2 activity worsens endothelial dysfunction through nitric oxide scavenging, pro-inflammatory signaling activation, and MMP stimulation, collectively increasing vascular permeability. Consistent with our earlier research [17], we found that OGD/R challenge significantly promoted expression of NOX-2, a key event in NOX activation that was effectively attenuated by TSI co-treatment. Moreover, rhPro-UK administration further potentiated p47phox membrane translocation, while TSI intervention restored its cytosolic localization, indicating specific regulatory effects of TSI on NOX-2 subunit trafficking under thrombolysis-associated stress conditions.

Collectively, the in vitro findings in HBMECs-demonstrating that TSI suppresses p47phox membrane translocation, reduces ROS overproduction, and prevents OGD/R+rhPro‑UK‑induced degradation of occludin and VE‑cadherin—provide a direct mechanistic basis for the in vivo observations. Specifically, this endothelial‑specific preservation of tight and adherens junction integrity translates into reduced paracellular leakage across the cerebral microvasculature. Accordingly, the significant decreases in Evans blue extravasation, FITC‑albumin leakage, and cerebral water content observed in rhPro‑UK + TSI‑treated mice can be attributed to TSI‑mediated stabilization of the endothelial barrier at the molecular level, rather than to secondary hemodynamic effects or alterations in thrombus resolution. This direct linkage between NOX-2‑derived oxidative stress, MMP‑2‑related gelatinolytic activity, junctional protein degradation, and macroscopic BBB breakdown is further supported by the concordant reduction in hemorrhagic transformation in the combination group. Thus, the cellular mechanism elucidated in HBMECs—inhibition of NOX-2 subunit membrane translocation and subsequent ROS/MMP‑2 cascade—directly explains the preservation of BBB integrity and the reduction of cerebral edema and hemorrhage observed in the animal model.

To validate that the inhibitory effect of TSI on NOX-2 ultimately mediates BBB protection through the NOX‑2‑ROS‑MMP‑2 axis, we introduced Vas2870, a specific NOX-2 inhibitor. Vas2870 significantly suppressed OGD/R‑induced gp91phox upregulation, ROS generation, and elevated MMP‑2 activity, with effects comparable to those of TSI. However, when TSI was combined with Vas2870, no further significant reduction in gp91phox expression or ROS generation was observed, suggesting that the mechanism of action of TSI is closely associated with gp91phox inhibition. This conclusion is supported by the fact that the NOX-2‑specific inhibitor Vas2870 functionally mimicked the effects of TSI, indirectly confirming the pivotal role of NOX-2 in the pharmacological actions of TSI.

This study offers a head-to-head comparison between rt-PA and rhPro-UK, establishing rhPro-UK’s superior safety profile in mitigating blood-brain barrier damage and hemorrhagic transformation following delayed thrombolysis. The combined use of TSI with rhPro-UK exhibits additive protective effects—preserving endothelial junction integrity, suppressing MMP‑2‑related gelatinolytic activity, and reducing oxidative stress—as evidenced in both in vivo and in vitro models. Limitations include the exclusive use of male mice, which constrains generalizability to female populations; a treatment window of 4.5 h that does not model ultra-late thrombolysis (beyond 6 h); insufficient investigation into pharmacokinetic interactions between TSI and rhPro-UK; and the absence of functional outcome assessments beyond 24 h.

In addition to endothelial dysfunction and tight junction disruption, some evidence indicates that pericytes and reactive astrocytes serve as core components of the neurovascular unit and jointly mediate BBB damage and neurological injury in ischemic stroke. Pericytes, as mural cells, stabilize the microvasculature and BBB by regulating endothelial junctions and secreting angiopoietin‑1 and PDGF‑BB through signaling axes. Ischemia induces their contraction, detachment, and apoptosis, resulting in capillary obstruction, hypoperfusion, and BBB leakage [31]. Astrocytes maintain vascular stability and barrier function, and after stroke, they undergo changes that can promote vascular repair. However, they can also produce factors that damage the vascular structure. Endothelial microvesicles regulate astrocyte functions and BBB integrity. Pericyte and astrocyte dysfunction contributes to stroke pathology by disrupting the neurovascular unit and amplifying endothelial damage [32, 33].

Because the BBB is composed of various cell types and the cellular functions during stroke are highly complex, this study could not encompass all cell types. Future research may explore these intricate functions through organoid culture methods in vitro, which represents a direction for our future studies.

Environmental factors (physical, chemical, and biological) are key modifiable risks for ischemic stroke. Short‑term exposure (within five days before stroke onset) to air pollutants (NO₂, SO₂, CO, O₃, PM₁, PM₂.₅, PM₁₀) is associated with higher incidence and mortality. PM₂.₅ is stably correlated with stroke onset, and NO₂ and SO₂ strongly influence mortality risk. Moreover, indoor chemical exposure from burning solid fuels also increases stroke risk [34]. Physical environmental factors such as temperature, housing, and water quality impact stroke risk. A poor living environment, including high PM₂.₅ exposure and inadequate water and heating, is linked to higher stroke risk. Extreme temperatures can cause physiological changes that increase the risk of ischemic stroke [35]. Acute infection is a trigger for ischemic stroke, with 10%‑35% of patients experiencing an infection within a week before stroke onset, peaking within 3 days post‑infection. Infections can cause systemic inflammation, endothelial damage, platelet activation, and a procoagulant state, leading to plaque instability and thrombosis. Stroke‑induced immunosuppression increases the risk of post‑stroke infection, worsening neurological outcomes and prognosis [36]. Environmental factors participate in the occurrence and progression of ischemic stroke through multiple pathways. Improving residential conditions and preventing infection are of great significance for the primary prevention of ischemic stroke.

In summary, this study demonstrates that rhPro-UK offers a safer thrombolytic profile than rt-PA by significantly reducing blood-brain barrier disruption and hemorrhagic transformation following delayed administration. Moreover, the combination of rhPro-UK with TSI provides additive cerebroprotection, effectively attenuating infarction, preserving endothelial junction integrity, and mitigating oxidative stress and MMP‑2‑related gelatinolytic activity. These findings support the potential clinical use of rhPro-UK, particularly in combination with TSI, as a promising strategy to improve safety and efficacy in delayed thrombolysis for ischemic stroke.

## Supplementary Information

Below is the link to the electronic supplementary material.

Supplementary Material 1

## Data Availability

Data are available on reasonable request.

## Abbreviations

AJs: Adherens junctions
BBB: Blood-brain barrier
BCA: Bicinchoninic Acid
CBF: Cerebral blood flow
DCFH-DA: 2’,7’-Dichlorodihydrofluorescein diacetate
EI: Extravasation indices
ELISA: Enzyme-linked immunosorbent assay
FITC: Fluorescein isothiocyanate
HBMEC: Human brain microvascular endothelial cells
HRP: Horseradish peroxidase
I/R: Ischemia-reperfusion
MCA: Middle cerebral artery
mNSS: modified Neurologic severity score
MMP: Matrix metalloproteinase
NOX: NADPH oxidase
OGD/R: Oxygen-glucose deprivation/reoxygenation
PBS: Phosphate-buffered saline
PVDF: Polyvinylidene fluoride
rhPro-UK: recombinant human prourokinase
ROS: Reactive oxygen species
rt-PA: recombinant tissue Plasminogen activator
TEER: Transendothelial electrical resistance
TCM: Traditional Chinese medicine
TJs: Tight junctions
TSI: Total salvianolic acid injection
TTC: 2,3,5-Triphenyltetrazolium chloride
vWF: von Willebrand factor
