# Supplementary material for: Adjuvant therapy with total salvianolic acid injection enhances the safety and efficacy of recombinant prourokinase in ischemic stroke by mitigating blood–brain barrier disruption
Source: J Transl Med. 2026 Jul 14;24:1272. doi: 10.1186/s12967-026-08583-3 (PMC13651226; doi:10.1186/s12967-026-08583-3)
Supplement: Supplementary file 1 [file 12967_2026_8583_MOESM1_ESM.docx]

**Supplemental Materials**

**Methods**

**Hematoxylin and Eosin (HE) and Masson's trichrome staining**

The experimental animal grouping was consistent with the manuscript Methods section. After collecting brain tissue, it was embedded in paraffin and sectioned. The HE staining procedure is as follows: Bake brain tissue sections at 65℃ for 30 min, dewax with xylene (2×20 min), rehydrate through graded ethanol, and rinse with tap water. Stain with hematoxylin (3-5 min), differentiate with 1% hydrochloric acid-ethanol, blue with Scott’s solution, counterstain with eosin (1-5 min). Dehydrate with graded ethanol, clear with xylene, and mount with neutral gum.

The Masson's trichrome staining procedure is as follows: Dewax and rehydrate paraffin sections. Optionally mordant in Bouin’s solution (56℃, 1 h), rinse. Stain with fresh Weigert’s hematoxylin (10 min), blue with warm tap water. Counterstain with Biebrich scarlet-acid fuchsin (10-15 min), differentiate with phosphotungstic-phosphomolybdic acid (15 min), stain with aniline blue (5-10 min), differentiate with 1% acetic acid. Dehydrate, clear and mount as above.

**Mouse model of thromboembolic stroke and drug administration**

Methods such as animal modeling, thrombus observation, TTC-stained brain tissue, and hemoglobin detection in tissues are described in the main manuscript. The key difference lies in the administration of intervention 1.5 hours after thrombus formation.

**Hepatic function, kidney function, coagulation function assay**

Four groups of normal mice were established: Control group (normal saline), TSI 1X group (the experimental dose used in this study, 15 mg/kg), TSI 5X group (five times the experimental dose), and TSI 10X group (ten times the experimental dose). All treatments were administered intravenously once daily for 7 consecutive days, after which plasma samples were collected for analysis. Hepatic, renal and coagulation function are measured using an automated biochemical analyzer (Chemray240/Chemray800/RAC-030, Rayto Life and Analytical Sciences Co., Shenzhen, China).

Hepatic function was comprehensively assessed by measuring serum levels of alanine aminotransferase (ALT), aspartate aminotransferase (AST), albumin (ALB), alkaline phosphatase (ALP), gamma-glutamyl transferase (GGT), total bilirubin (TBIL), direct bilirubin (DBIL), and total bile acid (TBA). These indicators collectively reflect hepatocellular injury, synthetic capacity, biliary excretion, and metabolic status.

Renal function was evaluated by measuring serum levels of urea (UREA), creatinine (CREA), and uric acid (UA), which together provide a reliable assessment of glomerular filtration and renal tubular function.

Coagulation function was assessed by measuring prothrombin time (PT), activated partial thromboplastin time (APTT), and fibrinogen (FIB), which comprehensively reflect coagulation homeostasis, bleeding tendency, and thrombotic risk.

**Figures and figure legends**

**Figure S1.** **Representative images of HE staining and Masson's trichrome staining for each experimental group.** (A) Representative image of HE staining, the scale bar in the upper panel is 1 mm, while the lower panel shows an enlarged view of the region of interest, scale bar is 250 μm; (B) Representative image of Masson tricolor staining: The scale bar in the upper panel is 1 mm, and the lower panel displays an enlarged view of the region of interest, scale bar is 250 μm.

**Figure S2.** **Thrombosis was treated with rt-PA and rhPro-UK thrombolysis respectively 1.5 hours after onset.** (A) Representative images for dynamically observing thrombus status at baseline, 15 min, 4.5 h, 5.5 h, and 28.5 h in every group (arrows indicate thrombus. bar=250 μm). (B) The statistical analysis of thrombus area for the above time points in mice (N=6). (C) Representative images of five coronal brain slices stained by TTC. Infarction was stained in white, while normal tissue was red. (D) Quantitative analysis of cerebral infarct volume (N=6). (E) Quantitative analysis of brain hemorrhage measured by colorimetric assay kit in each group.

**Figure S3.** **Effects of TSI continuous intravenous administration for 7 days on hepatic function, renal function and coagulation function.** (A) Hepatic function was assessed by measuring serum levels of ALT, AST, ALB, ALP, GGT, TBIL, DBIL, and TBA (N = 5 per group). (B) Renal function was evaluated by measuring serum levels of UREA, CREA, and UA (N = 5 per group). (C) Coagulation function was evaluated by measuring PT, APTT, and FIB (N = 5 per group).
